# Supplementary material for: Practices for preventing Hepatitis B infection among health science students in Ethiopia: Systematic review and meta-analysis
Source: PLoS One. 2024 Jul 10;19(7):e0306965. doi: 10.1371/journal.pone.0306965 (PMC11236149; doi:10.1371/journal.pone.0306965)
Supplement: S2 Table — (DOCX) [file pone.0306965.s002.docx]

**S2 Table: Quality assessment of Systematic Review and Meta-Analysis of Practices for Preventing Hepatitis B Infection among Health Science Students in Ethiopia.**

| References | **Theoretical approach** | | **Data collection** | **Study design** | **Validity** | | **Analysis** | | | | **Ethics** | | **Overall quality** | **First reviewer** | **Checker** |
| --- | --- | --- | --- | --- | --- | --- | --- | --- | --- | --- | --- | --- | --- | --- | --- |
|  | Is a qualitative approach appropriate? | Is the study clear in what it seeks to do? | How well was the data collection carried out? | How defensible/rigorous is the research design/methodology | Is the context clearly described? | Were the methods reliable? | Are the data ‘rich’? | Is the analysis reliable? | Are the findings convincing? | Are the conclusions adequate? | Was the study approved by an ethics committee? | Is the role of the researcher clearly described? |  |  |  |
| Allene et al | Appropriate | Clear | Adequately | Defensible | Clear | Reliable | Uncertain/not reported | Reliable | Convincing | Adequate | Yes | Yes | Medium | AAS | AAC |
| Abdela et al | Appropriate | Clear | Adequately | Defensible | Clear | Reliable | Rich | Reliable | Convincing | Adequate | Yes | not reported | Medium | GWK | AAC |
| Aynalem et al | Appropriate | Unclear | adequately | Not defensible | Clear | Uncertain | Poor | Uncertain | Not convincing | Inadequate | Not reported | Not clear | low | GWK | AAS |
| Gebremeskel et al | Appropriate | Clear | Adequately | Defensible | Clear | Reliable | Uncertain | Reliable | Convincing | Adequate | Yes | Uncertain | Medium | AAC | GWK |
| Mesfin et al | Appropriate | Clear | Adequately | Defensible | Clear | Reliable | Rich | Reliable | Convincing | Adequate | Yes | Not clear | Medium | ATD | GWK |
| Demsis et al | Appropriate | Unclear | adequately | Defensible | Clear | Uncertain | Poor | Uncertain | Not convincing | Inadequate | Not reported | Not clear | low | AAC | AAS |
| Haile et al | Appropriate | Clear | Adequately | Not defensible | Clear | Reliable | Uncertain | Reliable | Convincing | Adequate | Yes | Uncertain | Medium | AAS | GWK |

**Question codes:**

1. Was the sample frame appropriate to address the target population?

2. Were study participants sampled in an appropriate way?

3. Was the sample size adequate?

4. Were the study subjects and the setting described in detail?

5. Was the data analysis conducted with sufficient coverage of the identified sample?

6. Were valid methods used for the identification of the condition?

7. Was the condition measured in a standard, reliable way for all participants?

8. Was there appropriate statistical analysis?

9. was the response rate adequate, and if not, was the low response rate managed appropriately?
