# Supplementary material for: Practices for preventing Hepatitis B infection among health science students in Ethiopia: Systematic review and meta-analysis
Source: PLoS One. 2024 Jul 10;19(7):e0306965. doi: 10.1371/journal.pone.0306965 (PMC11236149; doi:10.1371/journal.pone.0306965)
Supplement: S3 Table — (DOCX) [file pone.0306965.s003.docx]

S3. Table

JBI Critical Appraisal Checklist for
systematic reviews and research syntheses

**Reviewer:** Adamu Ambachew Shibabaw and Alex Ayenew Chereka

**Date:** January 5, 2024

**Author:**  Gemeda Wakgari Kitil

**Year:** 2024, Record Number___4

|  | Yes | No | Unclear | Not applicable |
| --- | --- | --- | --- | --- |
| 1. Is the review question clearly and explicitly stated? | 🗹 | □ | □ | □ |
| 1. Were the inclusion criteria appropriate for the review question? | 🗹 | □ | □ | □ |
| 1. Was the search strategy appropriate? | 🗹 | □ | □ | □ |
| 1. Were the sources and resources used to search for studies adequate? | 🗹 | □ | □ | □ |
| 1. Were the criteria for appraising studies appropriate? | 🗹 | □ | □ | □ |
| 1. Was critical appraisal conducted by two or more reviewers independently? | 🗹 | □ | □ | □ |
| 1. Were there methods to minimize errors in data extraction? | 🗹 | □ | □ | □ |
| 1. Were the methods used to combine studies appropriate? | 🗹 | □ | □ | □ |
| 1. Was the likelihood of publication bias assessed? | 🗹 | □ | □ | □ |
| 1. Were recommendations for policy and/or practice supported by the reported data? | 🗹 | □ | □ | □ |
| 1. Were the specific directives for new research appropriate? | 🗹 | □ | □ | □ |

Overall appraisal: Include 🗹 Exclude □ Seek further info □

Comments (Including reason for exclusion)

________________________________________________________________________________________________________________________________________________________________________________________________________________________________________________________________________________________________
