# Supplementary material for: Practices for preventing Hepatitis B infection among health science students in Ethiopia: Systematic review and meta-analysis
Source: PLoS One. 2024 Jul 10;19(7):e0306965. doi: 10.1371/journal.pone.0306965 (PMC11236149; doi:10.1371/journal.pone.0306965)
Supplement: Attachment — Submitted filename: Point-by-point.docx [file pone.0306965.s006.docx]

**Dear Editor(s) of PLOS ONE Journal,**

We trust this message finds you well. We are pleased to submit the *first revision* of our manuscript titled "***Practices for Preventing Hepatitis B Infection among Health Science Students in Ethiopia: A Systematic Review and Meta-Analysis" (PONE-D-24-11778).***

We extend our sincere appreciation for your invaluable support throughout the review process. Your insightful comments and constructive feedback have been instrumental in refining our research. Your dedication to excellence in academic publishing is truly commendable.

We are deeply grateful for the thorough and constructive feedback provided by both the editors and reviewers. Your contributions have significantly enhanced the clarity, rigor, and overall quality of our work. The detailed suggestions and critical insights offered by the reviewers have been pivotal in addressing key areas and improving our manuscript substantially.

Thank you for considering our revised manuscript. We eagerly anticipate your feedback and remain available for any further revisions or clarifications.

Best regards,

**Corresponding Author**:

Gemeda Wakgari Kitil

[gemedawa425@gmail.com](mailto:gemedawa425@gmail.com)

# 1. Point-by-point response letter to Editor

**Dear Tebelay Dilnessa	Academic Editor of PLOS ONE journal,**

I hope this message finds you well. On behalf of my co-authors and myself, I would like to express our sincere gratitude for your invaluable efforts in handling our manuscript, "Practices for Preventing Hepatitis B Infection among Health Science Students in Ethiopia: A Systematic Review and Meta-Analysis." Your comments and suggestions have been incredibly helpful in refining our work. We greatly appreciate the time and expertise you dedicated to providing thorough and thoughtful feedback. Thank you once again for your guidance and support throughout the review process. Here is a point-by-point response addressing the changes made to your suggestions and comments:

**Editor**: Please submit your revised manuscript by Jul 07, 2024 11:59 PM. Please include the rebuttal letter for the response of the reviewer and editor, marked, and unmarked document when submitting your revised manuscript.

**Authors:** We sincerely appreciate your insightful comment. We have taken your feedback seriously and diligently addressed the concern you mentioned. Additionally, we have ensured that all necessary documents, as per your recommendation, have been submitted along with the revised manuscript within a given time. Thank you for guiding us through this process.

**Editor**: When submitting your revision, we need you to address these additional requirements.

**Authors**: Thank you for providing us with the additional requirements for our manuscript submission to PLOS ONE. We appreciate your thorough review and guidance to ensure compliance with the journal's style and submission standards. Below, we address each of the points raised:

**Editor comment-1**: Please ensure that your manuscript meets PLOS ONE's style requirements, including those for file naming

**Authors**: Thank you for your review and specific comments. We have carefully reviewed the PLOS ONE style requirements outlined in the provided templates and ensured that our manuscript adheres to these guidelines for file naming and formatting. Any necessary adjustments have been made to align our manuscript with the specified style. Please review the revised manuscript.

**Editor comment-2:** Kitil., et al, assessed the practices of health science students for preventing hepatitis B infection in Ethiopia. The way presented is very poor and requires a major revision grammatically, typographically, etc.

**Authors**: Thank you for your detailed feedback on our manuscript titled "Practices of Health Science Students for Preventing Hepatitis B Infection in Ethiopia: Systematic Review and Meta-Analysis. We appreciate your critical insights and are committed to improving the quality of our presentation. We acknowledge that the manuscript requires significant revisions in terms of grammar, typographical errors, and overall presentation. To improve the manuscript, we have thoroughly reviewed and corrected grammatical errors, rectify typographical mistakes, and enhance overall readability. Kindly review the revised manuscript.

**Editor comment-3:** The title "Systematic Review and Meta-Analysis of Practices for Preventing Hepatitis B Infection among Health Science Students in Ethiopia" It is better written, ‘Practices for Preventing Hepatitis B Infection among Health Science Students in Ethiopia: Systematic Review and Meta-Analysis’.

**Authors**: Thank you for your valuable feedback on our manuscript title. We appreciate your suggestion and agree that the revised title enhances clarity and readability. We have updated the title accordingly**.** See the revised manuscript

**Editor comment-4:** In the abstract part of conclusion, ‘Factors such as knowledge, age, needle stick injury exposure, and department influence these practices significantly.’ It does not give sense and needs revision. Again ‘ To improve prevention efforts, targeted interventions focusing on education and awareness are crucial. Addressing these disparities can lead to better infection control and public health outcomes in Ethiopia.’ It is a general concept and not emanated from the review result, so needs revision.

**Authors**: Thank you for your feedback. We have added appropriate captions for the supporting information files at the end of our manuscript and included the relevant files. We kindly invite you to review the revised manuscript, specifically **Page 2, lines 40-45**

**Editor comment-5:** In the result part of abstract and onwards, please replace the word ‘prevalence’ by ‘magnitude.’

**Authors**: Thank you for your invaluable comments and suggestions. We have replaced the word ‘prevalence’ with ‘magnitude’ in the abstract section as requested

**Editor comment-6:** In the result part of abstract ‘The statement ‘Factors significantly associated with these practices included good knowledge….’ It was vague and requires revision.

**Authors**: Thank you for your constructive feedback. We have revised the statement in the revised document.

**Editor comment-7:** In the methods part and the result part, your citations of figures were not correct. For example, (See S2 Table), (See Error! Reference source not found.), (See 9 Table 1), (see Figure 2), etc. Here, the word ‘see’ was not necessary

**Authors**: Thank you for bringing this to our attention. We apologize for the oversight in the citation format. We have revised the methods and results sections to ensure that the citations of figures are correctly formatted without the unnecessary use of the word 'see'. Please see the revised manuscript for further clarification.

**Editor comment-8:** Have you taken an ethical clearance? From whom? Is it necessary?

**Authors**: Thank you for your insightful suggestions and comments on ethical clearance. In response to your request, this study involves conducting a systematic review and meta-analysis, falling within the realm of secondary research where the data utilized is already published and publicly available. Given that the study strictly adheres to the Preferred Reporting Items for Systematic Reviews and Meta-Analysis (PRISMA) guidelines, which ensure transparency and rigor in reporting, ethical clearance was not deemed necessary. The primary focus of this research is to synthesize and analyze existing data, rather than directly involving human subjects or primary data collection. Therefore, ethical clearance was not sought nor required for this study.

**Editor comment-9:** The conclusion and recommendation were not targeted and specific

**Authors**: Thank you for your valuable feedback on our manuscript. We have carefully revised the conclusion and recommendation sections based on your comments to provide clearer, more focused statements. The conclusion now succinctly summarizes the key findings and their implications, and the recommendations offer actionable, specific suggestions. Please review the updated sections in our revised manuscript. See again the revised manuscript on pages **18 & 19,** especially on lines 4**30-449.**

**Editor comment-10:** You have to follow the manuscript writing protocol of PLOS One

**Authors**: Thank you for your feedback on our manuscript. We have carefully considered your comments and made the necessary revisions

**Editor comment-11:** Considering that the author will significantly revise the paper, major revision was recommended.

**Authors**: Thank you for your feedback and thorough evaluation of our work. We have carefully considered your comments and agree that significant revisions are necessary to improve the quality and clarity of the paper.

We are committed to addressing all the issues raised and undertaken a comprehensive revision to ensure that the manuscript meets the standards of the journal. We provided a detailed response to each of your suggestions and made the necessary changes accordingly.

We understand the importance of this process and assure you that we have made every effort to incorporate your feedback effectively. Thank you once again for your valuable input, and we look forward to submitting the revised manuscript in due course.

# 2. Point-by-point response letter to Reviewer-1

**Authors**: Thank you for your valuable feedback on our manuscript, "***Practices for Preventing Hepatitis B Infection among Health Science Students in Ethiopia: A Systematic Review and Meta-Analysis***." We greatly appreciate the time and effort you have invested in providing insightful comments and suggestions. We have thoroughly considered your feedback and made the necessary revisions. Below, we address each of the points raised:

**Reviewer-1 Comment-1**:

1. The writing lacks fluency and has serious issues with grammar that need to be addressed.
2. The document suffers from major coherence problems and contains redundant ideas

**Authors**: Thank you for your feedback. We acknowledge the concerns regarding the lack of fluency and grammar issues in the manuscript. We have revised the structure of sentences, conducted a thorough grammar check, and sought feedback to improve the overall quality before resubmitting. Your observation regarding coherence problems and redundant ideas in the document is duly noted. We have worked on enhancing the flow of ideas and eliminating any unnecessary repetition to improve the overall clarity and coherence of the content

**Reviewer-1 comment-2**: The article’s searching strategy (search string) is inaccurate. The authors failed to identify the key terms related to the PICO (Population, Intervention, Comparison, Outcome) for the study. Consequently, the search string yielded an excessively large number of articles (22,150 articles), which is incorrect. With a properly structured search string, the number of articles should have been limited to several hundred. One of the main reasons for this oversight is the failure to include the country name “Ethiopia” in the search string, despite the study being conducted in Ethiopia. The authors should rectify this issue by restricting the search string with the country name.

**Authors:** Thank you for your feedback on our article. We appreciate your insights regarding the search strategy and its alignment with the PICO framework.

Upon careful consideration, we acknowledge the importance of refining our search strategy to better align with the PICO elements. In response to your comment, we have revised our search string to ensure the inclusion of key terms related to the Population, Intervention, Comparison, and Outcome of our study. We invited you to review our revised manuscript pages **7 & 8 lines 173-179.**

Regarding your comment on the article's search strategy, we acknowledge the oversight in not including "Ethiopia" in the search string, which led to an excessive number of articles. We have rectified this by revising the search strategy to include "Ethiopia" as a key term, ensuring more focused and relevant search results aligned with the study's context.

**Reviewer-1 comment-3** On page 12, the authors stated, “The analysis, with an I2 value of 0.0% and a p-value of 0.926, revealed no substantial heterogeneity among the included studies.” However, the analysis conducted is not appropriate as it appears to be a subgroup analysis rather than a simple forest plot. Furthermore, even if the analysis was appropriate, it is unclear why the authors conducted further subgroup analysis (figure 4). Additionally, conducting subgroup analysis for a few included studies raises concerns about its appropriateness. The subgroup analysis lacks results for I2 and p-values due to the insufficient number of articles for the subgroup analysis. The authors should reconsider their analysis approach and provide a clear rationale for conducting subgroup analysis.

**Authors**: Thank you for bringing this to our attention. We apologize for the confusion regarding the analysis presented on page 12. There seems to have been a misunderstanding. The results mentioned in that section were derived from the pooled factors associated with preventive practice toward HBV infection, as depicted in Figure 4.

We did not conduct a subgroup analysis as indicated. The results provided were from the overall analysis of factors associated with preventive practices toward HBV infection. We understand the importance of clarity in reporting our analysis approach and rationale. We have revised the manuscript to provide a clearer explanation of the analysis conducted and the rationale behind it. Please refer to the revised tracked manuscript, ***page 14, line 321***, for further details.

**Reviewer-1 comment-4**: The references do not adhere to the journal’s guidelines. The formatting and style of the references are inconsistent. While most references are written as a list of the first author followed by “et al,” some references include the complete list of authors. The authors need to revise the references to ensure consistency and adherence to the journal's guidelines.

**Authors**: Thank you for your feedback on our manuscript. We appreciate your attention to detail regarding the references. We apologize for the inconsistency in formatting and style. We have revised the references to ensure they adhere to the journal's guidelines consistently.

Specifically, we have ensured that all references follow the prescribed format of listing the first author followed by "et al." where applicable. Additionally, we have rectified instances where the complete list of authors was mistakenly included. For further confirmation please refer to the revised tracked References ***pages 21 and 22, lines 509-end***, for details.

# 3. Point-by-point response letter to Reviewer-2

**Authors:** Thank you for your valuable feedback on our manuscript entitled "***Practices for Preventing Hepatitis B Infection among Health Science Students in Ethiopia: A Systematic Review and Meta-Analysis***." We appreciate the time you have taken to provide thoughtful comments and suggestions for improving our work. We have carefully considered your feedback and made the necessary revisions to address your comments. Below, we provide our responses to each of the points raised:

**Reviewer-2 Comment-1**: In the abstract and throughout the document, rather than using prevalence of practices, better replace it with magnitude.

**Authors**: Thank you for your valuable feedback and constructive suggestions. We have revised the abstract and the document as suggested. The term "prevalence of practices" has been replaced with "magnitude" to ensure clarity and precision throughout the manuscript. We appreciate your attention to detail and believe these changes enhance the overall quality of the paper. We invited you to review the entire revised manuscript.

**Reviewer-2 Comment-2:** Abstract (results sub-section), make the factors specific. Example, department (which department)…

**Authors**: Thank you for your insightful suggestions and feedback. We have revised the results section of the abstract to specify the departments more clearly. Please see the updated manuscript, especially the abstract and result section.

**Reviewer-2 Comment-3:** Is the protocol registered in PROSPERO?

**Authors**: Thank you for your inquiry. No, the protocol is not registered in PROSPERO. However, we have diligently adhered to stringent guidelines to guarantee transparency and reproducibility, as thoroughly outlined in our manuscript.

**Reviewer-2 Comment-4:** Include data extraction sheet and JBI tool as annexes

**Authors**: Thank you for your suggestion regarding the inclusion of the data extraction sheet and the JBI tool as annexes. We agree that providing these documents as annexes/Supporting information will enhance the transparency and reproducibility of our study. Accordingly, we have included the data extraction sheet and the JBI tool in the annexes/Supporting information of the revised manuscript. Please see the updated manuscript for additional information.

**Reviewer-2 Comment-5:** Data extraction and quality assessment are different. Do not mix up the statements in different section. Put the concepts in separate headings

**Authors**: Thank you for your valuable feedback. We apologize for any confusion caused by mixing data extraction and quality assessment in different sections. To address this, we have separated these concepts into distinct headings in the revised manuscript. This reorganization ensures clarity and enhances the overall structure of our work. We appreciate your insight and have made the necessary changes to improve the presentation.

**Reviewer-2 Comment-6:** Quality of the figures is poor

**Authors**: Your feedback regarding the quality of the figures is greatly appreciated. We acknowledge this concern and apologize for any inconvenience caused. We have promptly addressed this issue by revising the figures to ensure they are of high resolution and clarity in the revised version of the manuscript. Specifically, figures 1, 2, and 4 have been modified to meet the highest standards of quality. Additionally, we have verified that all figures are appropriately labeled and referenced for better comprehension. We are committed to enhancing the quality of the figures to improve the overall presentation of our work. Please see the updated manuscript for further clarification.

**Reviewer-2 Comment-7:** in the inclusion criteria, indicate the types of study designs considered

**Authors**: Thank you for your comment. In response, we have clarified the inclusion criteria to specify the types of study designs considered. Please refer to the updated manuscript, particularly ***page 7, lines 154-156***, for further details.

**Reviewer-2 Comment-8:** Outcome measurement is not clear. did you dichotomize practices of Health Science Students in preventing Hepatitis B infection? How did you do that for analysis? It has to be clear in the methods section

**Authors**: Thank you for your feedback; we appreciate your attention to detail. We would like to clarify that we did not dichotomize the practices of Health Science students in preventing Hepatitis B infection. This was an error in writing and a misunderstanding during manuscript preparation. We apologize for any confusion. Initially, I had written it as two main outcomes rather than dichotomizing it.

As indicated by the title of our manuscript, "Practices for Preventing Hepatitis B Infection among Health Science Students in Ethiopia: Systematic Review and Meta-Analysis," our study focuses on two main outcome measurements. The primary outcome of interest in this study examines the practices of Health Science students in preventing Hepatitis B infection, while the second outcome analyzes factors associated with these practices.

We have taken care to delineate these aspects in the methods section for better understanding. Specifically, for the first outcome, we quantify the practices using a detailed checklist. For the second outcome, we analyze associated factors by calculating the log odds ratio (OR) based on data from original articles. We invite you to review the updated manuscript on **page 10, lines 244-248.**

**NB:** In closing, on behalf of my co-authors, I want to extend our heartfelt gratitude to Editor and the reviewers for not only their roles as editors and reviewers but also for their invaluable guidance and mentorship throughout this process. Your feedback has not only improved the quality and clarity of our work but has also enriched our understanding of the subject matter. If there are any lingering questions or comments that require further attention, please don't hesitate to contact us. We are fully committed to addressing any outstanding concerns to the best of our ability. Once again, we sincerely appreciate your time, expertise, and mentorship.

Warm regards,

**Corresponding Author**:

Gemeda Wakgari Kitil

[gemedawa425@gmail.com](mailto:gemedawa425@gmail.com)
