# Supplementary material for: Practices for preventing Hepatitis B infection among health science students in Ethiopia: Systematic review and meta-analysis
Source: PLoS One. 2024 Jul 10;19(7):e0306965. doi: 10.1371/journal.pone.0306965 (PMC11236149; doi:10.1371/journal.pone.0306965)
Supplement: Attachment — Submitted filename: Point-by-point.docx [file pone.0306965.s007.docx]

**Dear Editor,** **Tebelay Dilnessa, MSc Academic Editor of PLOS ONE**

We trust this message finds you well. We are writing to express our deepest gratitude for your invaluable support and guidance in the editorial process of our manuscript. We are pleased to submit the second revision of our manuscript, titled “***Practices for Preventing Hepatitis B Infection among Health Science Students in Ethiopia: A Systematic Review and Meta-Analysis" (PONE-D-24-11778R1)***. We have no words to adequately thank you and truly appreciate your work.

Your detailed feedback, constructive criticism, and meticulous corrections have once again significantly enhanced the quality of the manuscript. We truly appreciate the time and effort you invested in providing such comprehensive and insightful comments. Your expertise and suggestions have not only improved the clarity and coherence of the content but also strengthened the overall argument and presentation of the study.

We are particularly grateful for your encouragement and the positive reinforcement you provided alongside your critical observations. This process has been a learning experience for us, and we are confident that the manuscript is now much more robust and impactful due to your contributions.

Thank you once again for your dedication and for helping us bring our research to its best possible form. We appreciate your consideration of our revised manuscript. We eagerly anticipate your feedback and remain available for any further revisions or clarifications.

Best regards,

**Corresponding Author**:

Gemeda Wakgari Kitil

[gemedawa425@gmail.com](mailto:gemedawa425@gmail.com)

# 1. Point-by-point response letter to Editor

**Dear Editor,**

Once again, we greatly appreciate the time and expertise you dedicated to providing thorough and thoughtful feedback. We provide a point-by-point response addressing the changes based on your suggestions and comments as follows:

**Editor**: Please submit your revised manuscript by Aug 02, 2024 11:59 PM. Please include the rebuttal letter for the response of the reviewer and editor, marked, and unmarked document when submitting your revised manuscript.

**Authors:** Thank you for your insightful comments. We have diligently addressed your feedback and included all required documents with the revised manuscript on time to prevent any delays.

**Editor**: When submitting your revision, we need you to address these additional requirements.

**Authors**: Thank you for providing us with the additional requirements for our manuscript submission to PLOS ONE. We appreciate your thorough review and guidance to ensure compliance with the journal's style and submission standards. Below, we address each of the points raised:

**Editor comment-1**: Journal Requirements:

Please review your reference list to ensure that it is complete and correct. If you have cited papers that have been retracted, please include the rationale for doing so in the manuscript text, or remove these references and replace them with relevant current references. Any changes to the reference list should be mentioned in the rebuttal letter that accompanies your revised manuscript. If you need to cite a retracted article, indicate the article’s retracted status in the References list and also include a citation and full reference for the retraction notice.

**Authors**: Thank you for your instructions regarding the reference list. We have reviewed our manuscript, and no retracted articles were cited. Therefore, no changes were necessary in this regard. We appreciate your attention to this matter.

**Editor comment-2:** Line 28: ……………………and the I2 statistic. Write as,………… and the I2 statistic.

**Authors**: Thank you for your detailed feedback and critical insights. We are committed to improving the quality of our presentation. In response to your comment, we have reviewed the entire manuscript and replaced "I2" with “I^2”^. We kindly invite you to review the updated manuscript for further clarification.

**Editor comment-3:** Please make the heading and subheading writing style uniform. For example, 3. Results; 3.1. Study selections; 3.2 Characteristics of included studies for review; 3.4 Publications bias; Funding:; Acknowledgments:, etc. The use of full stop and other punctuation marks create the confusion.

**Authors**: Thank you for your feedback and suggestions regarding the uniformity of heading and subheading writing style in our manuscript. We have carefully reviewed your comments and made the necessary adjustments to ensure consistency throughout. We have addressed the issue of punctuation marks to eliminate confusion and maintain clarity across headings and subheadings. We kindly invite you to see the updated manuscript.

**Editor comment-4:** In the tables’ description, the year (2024) better be at the end of the statement if the year is necessary. For example, Table 1.

**Authors**: Thank you for your suggestion regarding the tables' descriptions. We have revised them accordingly, placing the year (2024) at the end of each statement, as recommended. Please find the updated tables in our revised manuscript. We kindly invite you to review the revised manuscript, specifically **Page 10, line 241**

**Editor comment-5:** What is the unit/measurement of the last column of Table 1 (Practice column).’

**Authors**: Thank you for your invaluable comments and suggestions. The unit of measurement for the last column (Practice column) in Table 1 is presented as "Percentage (%)". Each value represents the percentage of respondents or instances for each practice category. Review the updated manuscript.

**Editor comment-6:** Line 212-216: The subheading, ‘2.8 Patient and Public Involvement’ together with its content was not necessary. So please remove it.

**Authors**: Thank you for your constructive feedback. Regarding your comment on lines 212-216, we have made the necessary revisions. Specifically, we have removed the subheading "2.8 Patient and Public Involvement" as you suggested. We appreciate your thorough review and believe this change improves the clarity and structure of the manuscript.

**Editor comment-7:** For the discussion; you have to use similar articles (SRMA) for compare and contrast and then justify it.

**Authors**: Thank you for your suggestion regarding the discussion section. We have incorporated comparable systematic review and meta-analysis (SRMA) articles for contrast and justification, as per your advice. However, due to the scarcity of literature on systematic review and meta-analysis with the same topic, we prefer to discuss them alongside similar articles/primary article rather than systematic review and meta-analysis.

**Editor comment-8:** Your conclusion and recommendation were too ambitious. It should be specific and based on your result.

**Authors**: Thank you for your valuable feedback and suggestions. We have carefully reconsidered our conclusion and recommendations to ensure they are specific and grounded in our findings. We incorporated the revised conclusion and recommendations in the updated manuscript; especially on pages 15 and 16 lines 344-377.

**Editor comment-9:** In the ‘Strengths and limitations of the study’. Select the most important strength and limitations only; avoid unnecessary concepts and details.

**Authors**: Thank you for your valuable feedback on our manuscript. Based on your comments, we have revised the Strengths and Limitations sections to focus on essential aspects while avoiding unnecessary details. Please review the updated sections in our revised manuscript, particularly on page **16, lines 391-397,** for clearer, more focused statements**.**

**Editor comment-10:** Line 375: ‘Availability of Data and Materials:’ It is better written as, ‘Availability of data’

**Authors**: Thank you for your feedback on our manuscript. We have carefully considered your comments and made the necessary revisions to the updated manuscript and corrected it as “Availability of Data and Materials” was replaced with “‘Availability of data’.

**Editor comment-11:** Write properly reference number 2 based on the standard.

**Authors**: Thank you for your feedback. In response to your Comment, we have revised reference number 2 to comply with the standard citation format. We appreciate your attention to detail and have ensured that the reference is now correctly formatted.

**Editor comment-12:** Figure 1 is still lacking clarity; the decoration makes this table unclear. Prepare it clearly please again.

**Authors**: Thank you for your feedback on Figure 1. We acknowledge that the current version lacks clarity due to excessive decoration. We have revised Figure 1 to improve its clarity by removing unnecessary decorative elements and ensuring that the information is presented in a straightforward and comprehensible manner. Please find the updated figure attached for your review.

**NB.**

If there is any further information needed regarding this manuscript, please feel free to ask. We are ready to respond to your request. Additionally, we would appreciate your feedback on any revisions required or any comments that have not been fully addressed.

Thank you for your attention to this matter.

Best regards,
