# Supplementary material for: Practices for preventing Hepatitis B infection among health science students in Ethiopia: Systematic review and meta-analysis
Source: PLoS One. 2024 Jul 10;19(7):e0306965. doi: 10.1371/journal.pone.0306965 (PMC11236149; doi:10.1371/journal.pone.0306965)
Supplement: Attachment — Submitted filename: Point-by-point-3.docx [file pone.0306965.s008.docx]

**Dear Editor,** **Tebelay Dilnessa,**

**Academic Editor of PLOS ONE**

We are truly grateful for your unwavering support and guidance throughout the editorial journey of our manuscript titled “***Practices for Preventing Hepatitis B Infection among Health Science Students in Ethiopia: A Systematic Review and Meta-Analysis" (PONE-D-24-11778R2).*** It is with great pleasure that we submit the third revised version of our manuscript to you.

Your meticulous feedback, constructive criticism, and insightful corrections have not only refined the manuscript but also enriched its depth and impact. Your expertise has played a crucial role in improving the clarity and coherence of our study, and we deeply appreciate the time and effort you have dedicated to this process.

We are particularly grateful for your encouragement and positive reinforcement alongside your thorough critiques. This collaborative effort has been an enlightening experience for us, and we are confident that the manuscript is now stronger and more compelling thanks to your invaluable contributions.

Thank you once again for your dedication in helping us shape our research into its best possible form. We eagerly await your feedback on this revised submission and remain available for any further adjustments or clarifications you may require.

Warm regards,

**Corresponding Author**: Gemeda Wakgari Kitil

[gemedawa425@gmail.com](mailto:gemedawa425@gmail.com)

# 1. Point-by-point response letter to Editor

**Dear Editor,**

We sincerely appreciate the thorough and thoughtful feedback you've provided once again. In response to your suggestions, we have meticulously revised our manuscript. Below is our detailed response addressing the changes:

**Editorial Deadline:** Please submit your revised manuscript by Aug 08, 2024, 11:59 PM, including the rebuttal letter and both marked and unmarked document versions.

**Authors:** Thank you for your insightful comments. We have promptly incorporated all necessary documents with our revised submission to ensure timely processing.

**Editor comment-1**: Journal Requirements:

Please review your reference list to ensure that it is complete and correct. If you have cited papers that have been retracted, please include the rationale for doing so in the manuscript text, or remove these references and replace them with relevant current references. Any changes to the reference list should be mentioned in the rebuttal letter that accompanies your revised manuscript. If you need to cite a retracted article, indicate the article’s retracted status in the References list and also include a citation and full reference for the retraction notice.

**Authors**: Thank you for your guidance on the reference list requirements. We have thoroughly reviewed our manuscript and confirmed that no retracted articles were cited. Therefore, no changes were necessary in this regard. We appreciate your attention to this matter.

**Editor comment-2:** Line 103: 2. Methods and materials; It is better written as, ‘2. Materials and Methods’.

**Authors**: Thank you for your valuable feedback. We have revised the section title from "2. Methods and materials" to "2. Materials and Methods" to adhere to conventional scientific writing standards. This change should enhance the clarity and readability of the document. We kindly invite you to review the updated manuscript on **page 5** for further clarification.

**Editor comment-3:** Comment from previous session: Please make the heading and subheading writing style uniform. For example, 3. Results; 3.1. Study selections; 3.2 Characteristics of included studies for review; 3.4 Publications bias, etc. The use of full stop and other punctuation marks create the confusion.

**Authors**: Thank you for your feedback. We would like to say sorry for the previously unaddressed comment. We have reviewed the document and made the necessary adjustments to ensure consistency in the headings and subheadings. They now follow a uniform style without unnecessary punctuation marks, such as full stops, which should enhance readability and clarity throughout the manuscript.

**Editor comment-4:** In the recommendation part remove the subheadings (Education and Awareness Programs, Training on Safe Practices, Curriculum Integration, Support Services and Policy Development.

**Authors**: Thank you for your suggestion. We have revised the recommendation section by removing the subheadings (Education and Awareness Programs, Training on Safe Practices, Curriculum Integration, Support Services, and Policy Development). This change aims to streamline the presentation and ensure a more cohesive flow of recommendations. We kindly invite you to review the revised manuscript, specifically **Page 15, lines 346-362**

**Editor comment-5:** Line 378: Declaration of Non-Competing Interests: First avoid the use of colon mark (:). It is better written as, Competing interests: The authors have declared that no competing interests exist.

**Authors**: Thank you for your comment. We have revised the statement as follows: "Competing interests: The authors have declared that no competing interests exist." This adjustment removes the colon and maintains clarity regarding our declaration of non-competing interests. We invite you to review the updated manuscript.

**Editor comment-6:** The order of numbering better be removed from (6-8), but leave reference numbering and make it; ‘6. References’.

**Authors**: Thank you for your constructive comments and suggestions. We have removed the numbering from (6-8) and retained the reference numbering, now titled “**6. References**”. This adjustment aims to improve clarity and consistency in the document structure. We kindly invite you to see the revised manuscript.

**Editor comment-7:** You can remove the ‘Authors’ contribution’ as the system creates it.

**Authors**: Thank you for your input. We've removed the 'Authors' contribution' section as it is automatically generated by the system, ensuring a more streamlined presentation of the document.

**Editor comment-8:** Please revise all the references. For example, references such as 11, 12, 14, 17, 19, 20, 21, 23, 24 are written correctly as follows.

**Authors**: Thank you for highlighting the need for revisions to our references. We have carefully reviewed and corrected all references as per your instructions. The revised manuscript now accurately reflects the correct formatting for each cited source. In the tracked manuscript version, references 23, 26, 27, and 31 were highlighted in red color. These references were duplicates of the revised references and have been removed in the cleaned manuscript.
